# Supplementary material for: Clinical predictors of mortality in patients with pseudomonas aeruginosa infection
Source: PLoS One. 2023 Apr 28;18(4):e0282276. doi: 10.1371/journal.pone.0282276 (PMC10146515; doi:10.1371/journal.pone.0282276)
Supplement: Attachment — Submitted filename: Comments to the Author.docx [file pone.0282276.s001.docx]

**Comments to the Author**

1. Is the manuscript technically sound, and do the data support the conclusions?

The manuscript must describe a technically sound piece of scientific research with data that supports the conclusions. Experiments must have been conducted rigorously, with appropriate controls, replication, and sample sizes. The conclusions must be drawn appropriately based on the data presented.

Reviewer #1: Yes

Reviewer #2: Yes

2. Has the statistical analysis been performed appropriately and rigorously?

Reviewer #1: Yes

Reviewer #2: Yes

3. Have the authors made all data underlying the findings in their manuscript fully available?

The [PLOS Data policy](https://eur03.safelinks.protection.outlook.com/?url=http%3A%2F%2Fwww.plosone.org%2Fstatic%2Fpolicies.action%23sharing&data=05%7C01%7Czk10%40aub.edu.lb%7C2da513dd98014575cacb08daec913e2e%7Cc7ba5b1a41b643e9a1206ff654ada137%7C1%7C1%7C638082405805200168%7CUnknown%7CTWFpbGZsb3d8eyJWIjoiMC4wLjAwMDAiLCJQIjoiV2luMzIiLCJBTiI6Ik1haWwiLCJXVCI6Mn0%3D%7C3000%7C%7C%7C&sdata=crVbKYNELmn1yn1e3DMGcrKFedau0ssOxnMsoLrRvC4%3D&reserved=0) requires authors to make all data underlying the findings described in their manuscript fully available without restriction, with rare exception (please refer to the Data Availability Statement in the manuscript PDF file). The data should be provided as part of the manuscript or its supporting information, or deposited to a public repository. For example, in addition to summary statistics, the data points behind means, medians and variance measures should be available. If there are restrictions on publicly sharing data—e.g. participant privacy or use of data from a third party—those must be specified.

Reviewer #1: Yes

Reviewer #2: Yes

4. Is the manuscript presented in an intelligible fashion and written in standard English?

PLOS ONE does not copyedit accepted manuscripts, so the language in submitted articles must be clear, correct, and unambiguous. Any typographical or grammatical errors should be corrected at revision, so please note any specific errors here.

Reviewer #1: Yes

Reviewer #2: Yes

5. Review Comments to the Author

Please use the space provided to explain your answers to the questions above. You may also include additional comments for the author, including concerns about dual publication, research ethics, or publication ethics. (Please upload your review as an attachment if it exceeds 20,000 characters)

Reviewer #1: The overall manuscript is very well written, detailed and well explained. The data is presented well, the literature review is lucid, and the figures are very clear. However, I would like to recommend minor revisions for the manuscript.
1. Abstract --" Pseudomonas aeroginosa" should be in italics.
2. Introduction --" Intra-abdominal" should be hyphenated.
3. Materials and Methods -- Please remove the term " Definitions". Also P. aeroginosa should be in italics.

**Thank you for your comments.**

**We will apply all the proposed suggestions.**

Reviewer #2: Pseudomonas aeruginosa is a relevant microorganism associated with severe infections, mostly in hospitalized persons. The manuscript is about predictors of mortality in patients with P aeruginosa infections in Lebanon.
The study has an understandable designed but I miss information concerning methodology and data

The study was a cross-sectional one, involving 196 patients from 2017 to 2020 with positive cultures for P aeruginosa who developed an infection.
I wonder how infections were diagnosed as the differential diagnosis between infection and colonization is not always easy. And how many patients with a first colonization became infected?

**Thank you for pointing this out. Patients were considered infected if they displayed typical infection symptoms, such as fever, a positive culture, and symptoms related to the infection site. Also, taking into consideration the daily infectious diseases notes. If any doubt, another study researcher would reassess the eligibility of the patient.**

Acertement of the inf vs conzation status was made based on f=nf dis consultation sheet.(metods)

The authors enroled in the same study patients in hospitalization (50%), outpatients (25%) and health care associated (25%) patients. In the later group, the infection was treated where?
I wonder if this heterogeneity of the group cannot give be responsible for differences from other studies in the literature, concerning for instance prevalence of MDR isolates

- **Thank you for your response. To clarify, we classified the infection of all included hospitalized patients into hospital-acquired, community-acquired, and healthcare. Healthcare-associated infection was defined as an infection occurring in patients receiving home and/or ambulatory, intravenous therapy, chemotherapy, hemodialysis, wound care, specialized nursing care, or who had attended a hospital clinic within the last 30 days; patients hospitalized in an acute care hospital for ≥ 2 days within the last 90 days; and those residing in a nursing home or long-term care facility. we did not include outpatients only in-patients. The prevalence of MDR *P. aeruginosa* was 10%, which is consistent with previous data from our center**

I would like to have more information about the type of clinical infection, as this is a clinical study. What about the health-care infections for instance?

In 24% of cases P aeruginosa was not isolated from the blood, respiratory specimes or urine. From where the strains were isolated? At what kind of infections were they associated?

**Thank you for your comment. P. aeruginosa isolates were recovered most frequently from respiratory specimens (48.0%), followed by urine (20.3%), and bloodstream (7.7%), and in 24.0% of cases, the organism was isolated from various other sources like wounds, stool, axillary((!!colonization)**

ICU acquired infections were more severe than the others? And how many patients were in the need of IcU admission?

**Thank you. As reported in table 4, 53/ 196 (27%) of patients required ICU admission. The most complications encountered were the acquisition of a hospital-acquired infection other than the index infection (28.1%), admission to the intensive care unit (27.0%), sepsis (26.0%), recurrent infection (21.2%), and septic shock (18.9).**
What were the criteria for a dual antipseudomonal therapy?

**Thank you for your comment. We don’t interfere with the treatment plan as it is a retrospective study. However, according to the guidelines, patients were treated with dual anti-pseudomonal therapy if MDR, severe sepsis, neutropenic patients with bacteremia, burn patients with serious infections, and severe pneumonia in ICU.**

Finally some remarks

a) Abstract
“Pseudomonas aeruginosa (PSA) is difficult to treat microorganism..”- in spite of “the infection caused by PSA is difficult to treat..”

**Thank you. We modified it.**

b) Page 16 - “Similar findings were reported in.” need to be finished the sentence

**Thank you. We removed the sentence because it’s a duplicate.**

6. PLOS authors have the option to publish the peer review history of their article ([what does this mean?](https://eur03.safelinks.protection.outlook.com/?url=https%3A%2F%2Fjournals.plos.org%2Fplosone%2Fs%2Feditorial-and-peer-review-process%23loc-peer-review-history&data=05%7C01%7Czk10%40aub.edu.lb%7C2da513dd98014575cacb08daec913e2e%7Cc7ba5b1a41b643e9a1206ff654ada137%7C1%7C1%7C638082405805200168%7CUnknown%7CTWFpbGZsb3d8eyJWIjoiMC4wLjAwMDAiLCJQIjoiV2luMzIiLCJBTiI6Ik1haWwiLCJXVCI6Mn0%3D%7C3000%7C%7C%7C&sdata=t%2FTqIH6P6jUaJbhRJyrvDru4BFpo%2FTj6%2FYu3jJM3%2Buk%3D&reserved=0)). If published, this will include your full peer review and any attached files.


If you choose “no”, your identity will remain anonymous but your review may still be made public.


**Do you want your identity to be public for this peer review?** For information about this choice, including consent withdrawal, please see our [Privacy Policy](https://eur03.safelinks.protection.outlook.com/?url=https%3A%2F%2Fwww.plos.org%2Fprivacy-policy&data=05%7C01%7Czk10%40aub.edu.lb%7C2da513dd98014575cacb08daec913e2e%7Cc7ba5b1a41b643e9a1206ff654ada137%7C1%7C1%7C638082405805200168%7CUnknown%7CTWFpbGZsb3d8eyJWIjoiMC4wLjAwMDAiLCJQIjoiV2luMzIiLCJBTiI6Ik1haWwiLCJXVCI6Mn0%3D%7C3000%7C%7C%7C&sdata=AbyDv5qj6Q%2FiRWOdBoSxbWeL%2F0BElWTSodtFBq0k7z0%3D&reserved=0).

Reviewer #1: No

Reviewer #2: No
