# Supplementary material for: Clinical predictors of mortality in patients with pseudomonas aeruginosa infection
Source: PLoS One. 2023 Apr 28;18(4):e0282276. doi: 10.1371/journal.pone.0282276 (PMC10146515; doi:10.1371/journal.pone.0282276)
Supplement: Attachment — Submitted filename: Response to Reviewers 2.docx [file pone.0282276.s002.docx]

**Response to Reviewers’ Comments**

Reviewer #2:

Please explain "healthcare-associated" infections in the text as hospital acquired infections are also health care associated - you did it out of the paper, please see answers to reviewers.

**Thank you for pointing this out. We added the following definitions:**

**Infections were considered community-acquired if they manifested outside the hospital or were diagnosed within 48 hours of admission without any healthcare encounter in the past 30 days.**

**Hospital-acquired infection was defined as an infection that manifested 48 hours or more after hospital admission or within 7 days of discharge.**

**Healthcare-associated infection was defined as an infection occurring in patients receiving home and/or ambulatory** [**intravenous therapy**](https://www.sciencedirect.com/topics/medicine-and-dentistry/intravenous-drug-administration)**, chemotherapy,** [**hemodialysis**](https://www.sciencedirect.com/topics/medicine-and-dentistry/hemodialysis)**, wound care, specialized nursing care, or who had attended a hospital clinic within the last 30 days; patients hospitalized in an acute care hospital for ≥ 2 days within the last 90 days; and those residing in a nursing home or long-term care facility.**

2.Please note that the place of isolation is not the same of type of infection - we may have a pyelonephritis or a simple ITU from a positive urine culture, this should be clear, in all the results

**Thank you, we replaced the “site of infection” phrase with “type of infection” throughout the text.**

3.I wonder from the 24 samples out of the most common places of isolation how many cases from cerebrospinal samples were positive, and what was the course of the disease - this is a quite rare situation would be important to detail.

**Thank you for your comment.** **We had one CSF sample from a lymphoma patient who had a good outcome. We will be happy to add more details to the text if so required.**

4.Please answer the question about infections acquired in ICU - were they different? ICU acquired infections were more severe than the others? And how many patients were in the need of ICU admission?

**Thank you for your comments.**

**On bivariable analysis, we found that ICU admission was associated with a worse outcome (mortality). However, this relationship was no longer significant when we adjusted for confounders as evidenced by the regression analysis in Table 4. A total of 53/196 (27%) patients required ICU admission. With a p-value of 0.011, ICU mortality was 17/55 (30.9%) and non-ICU mortality was 21/196 (17.5%).**

**We have added this information to the text**.

Please correct in
line 79 -cut "Written consents taken**" done**
line 149 ("in" in spite of "is") ?? **done**

and 164 (confounders "4") **done**
